# The Role of Mass Spectrometry in Structural Studies of Flavin-Based Electron Bifurcating Enzymes

**DOI:** 10.3389/fmicb.2018.01397

**Published:** 2018-07-05

**Authors:** Monika Tokmina-Lukaszewska, Angela Patterson, Luke Berry, Liam Scott, Narayanaganesh Balasubramanian, Brian Bothner

**Affiliations:** Department of Chemistry and Biochemistry, Montana State University, Bozeman, MT, United States

**Keywords:** chemical cross-linking, hydrogen deuterium exchange, protein labeling, native mass spectrometry, electron bifurcation, protein structure, protein-modeling, mass spectrometry

## Abstract

For decades, biologists and biochemists have taken advantage of atomic resolution structural models of proteins from X-ray crystallography, nuclear magnetic resonance spectroscopy, and more recently cryo-electron microscopy. However, not all proteins relent to structural analyses using these approaches, and as the depth of knowledge increases, additional data elucidating a mechanistic understanding of protein function is desired. Flavin-based electron bifurcating enzymes, which are responsible for producing high energy compounds through the simultaneous endergonic and exergonic reduction of two intercellular electron carriers (i.e., NAD^+^ and ferredoxin) are one class of proteins that have challenged structural biologists and in which there is great interest to understand the mechanism behind electron gating. A limited number of X-ray crystallography projects have been successful; however, it is clear that to understand how these enzymes function, techniques that can reveal detailed in solution information about protein structure, dynamics, and interactions involved in the bifurcating reaction are needed. In this review, we cover a general set of mass spectrometry-based techniques that, combined with protein modeling, are capable of providing information on both protein structure and dynamics. Techniques discussed include surface labeling, covalent cross-linking, native mass spectrometry, and hydrogen/deuterium exchange. We cover how biophysical data can be used to validate computationally generated protein models and develop mechanistic explanations for regulation and performance of enzymes and protein complexes. Our focus will be on flavin-based electron bifurcating enzymes, but the broad applicability of the techniques will be showcased.

## Introduction

Electron bifurcation, which simultaneously sends electrons along high- and low-energy paths within the same protein complex, is an important mechanism for biological energy conservation. Electron bifurcation was first detailed in the Q-cycle, which is part of the aerobic respiratory chain (Mitchell, 1975). In contrast to the quinone-based electron bifurcation found in the Q-cycle, flavin-based electron bifurcation (FBEB) is used by anaerobic microorganisms to generate high energy compounds such as hydrogen gas and reduced ferredoxin (Herrmann et al., 2008; Li et al., 2008; Buckel and Thauer, 2013, 2018a,b; Peters et al., 2016). By coupling exergonic and endergonic oxidation–reduction reactions, electron bifurcation is able to circumvent thermodynamic barriers and minimize free energy loss; in other words, electron bifurcation maximizes the efficiency of biological energy conversion. These enzymes have evolved sophisticated ways to control the flow of electrons known as electron gating, which prevents both electrons in a pair from traveling down the exergonic pathway. Several systems capable of undergoing FBEB have been identified in recent years, such as the NADH-dependent ferredoxin-NADP^+^ oxidoreductase (Nfn), the electron-transferring flavoprotein (Etf), and, as is proposed, the [FeFe]-hydrogenase (Hyd) (Schut and Adams, 2009; Schuchmann and Müller, 2012; Wang et al., 2013; Buckel and Thauer, 2018a). These protein complexes vary in number of subunits and cofactor type and content. A common feature of all enzymes capable of FBEB is a central flavin molecule, either flavin mononucleotide (FMN) or flavin adenine dinucleotide (FAD) that directs electrons from medium potential donors along two different pathways. One is a high potential path that ends in an endergonic reduction reaction. The other path has a low potential, ending with an exergonic reduction reaction. Electrons arrive at their designated reaction centers (low- and high-potential acceptors) through an electron conduit, chain of iron-sulfur ([Fe–S]) clusters or other flavin molecules located in a protein complex or coupled to other enzymes [such as the butyryl-coenzyme A (CoA) dehydrogenase (Bcd) forming the Etf-Bcd complex]. The beauty of electron bifurcation is the production of highly reduced compounds, such as ferredoxin (Fd) and flavodoxin (Fld), from lower potential compounds without using the hydrolysis of high energy nucleotide phosphates such as ATP. Fd and Fld are responsible for providing electrons for high-energy reactions such as nitrogen fixation.

Enzymes employing FBEB are timely subjects for studies on the evolution of early life processes including aerobic respiration, control of electron flow in metabolism, and understanding proton coupled electron transfer reactions in biological systems (Herrmann et al., 2008; Buckel and Thauer, 2013; Peters et al., 2016). Details on fundamental concepts, challenges in research and future directions in the field of electron bifurcation can be found in these recent reviews: Buckel and Thauer (2013, 2018a,b), Metcalf (2016), and Peters et al. (2016).

A great deal of work has gone into investigating the mechanism of FBEB. This work has employed spectroscopic, electrochemical, and kinetic approaches. However, since the discovery of FBEB there has been a major push for determining the 3D structures of the protein complexes. This is because structural characterization lays the foundation for mechanistic studies of how bifurcation and electron gating are performed, which is crucial for the development of biotechnological adaptations for efficient and sustainable alternative fuel production.

Traditionally, X-ray crystallography, cryo-electron microscopy (cryo-EM) and nuclear magnetic resonance spectroscopy (NMR) are the favored techniques for developing high-resolution 3D structures of proteins. X-ray crystallography has been successfully applied to FBEB enzymes, specifically the Etf-butyryl-CoA dehydrogenase (EtfAB-Bcd), the NADH-dependent ferredoxin:NADP^+^ oxidoreductase (NfnI and II) from *Thermotoga maritima* and *Pyrococcus furiosus*, and the caffeyl-CoA reductase (CarABC) from *Acetobacterium woodii* (Chowdhury et al., 2014; Demmer et al., 2016, 2017, 2018; Lubner et al., 2017; Nguyen et al., 2017). These studies have allowed for the proposal of a detailed mechanism for electron bifurcation within the Nfn system. An alternative technique for structural determination is cryo-EM (Bai et al., 2015). The main advantage of using cryo-EM is that the protein is flash-frozen. This allows for the in-solution conformation of a protein or protein complex to be captured, membrane associated domains to be identified, and the resolution is such that amino acid side chains and metal clusters can be fit within the electron density (Zhang et al., 2003; Nogales, 2015). Cryo-EM was originally developed to look at large mega-dalton complexes such as viruses (Zhang et al., 2003), and while recent advancements have made it possible to apply it to smaller complexes, the FBEB enzymes are still on the lower end of the cryo-EM molecular weight range (Bai et al., 2015).

One of the greatest challenges in studying the FBEB enzymes is that the majority of the systems are rapidly inactivated in the presence of even trace amounts of oxygen. The presence of oxygen can cause [Fe-S] clusters to change conformation in an irreversible process, which leads to improper protein folding and, eventually, to loss of functionality by the entire complex (Khoroshilova et al., 1997). The rule of thumb is that the higher the number of [Fe-S] clusters the higher the sensitivity to oxygen. Even though Etf systems that contain only flavin cofactors, such as EtfAB and EtfAB-Bcd (Aboulnaga et al., 2013; Chowdhury et al., 2014) are not considered oxygen sensitive, specific experimental design may include the use of flavin in the reduced state. Therefore, when required, all experiments must be carried out in strictly controlled, anaerobic conditions. Another challenge is that FBEB enzymes are multisubunit protein complexes containing multiple electron transfer centers such as [Fe-S] clusters and flavins (FAD or FMN). Because the oxidation state of these cofactors influences protein conformation and dynamics, FBEB enzymes pose a significant analytical challenge due to the number of possible ensembles present in solution. The sophistication of experimental design and the complexity of analysis increases with the number of subunits within a complex.

In the early discovery phase of the electron bifurcation field, the most commonly applied types of the mass spectrometry were inductively coupled plasma (ICP) and matrix-assisted laser desorption/ionization time of fight mass spectrometry (MALDI-TOFMS). ICP has been primarily used for cofactor analysis, which determines the type of metal and overall metal content (Verhagen et al., 1999; Mock et al., 2014). In addition, Chowdhury et al. (2016) have used ICP to demonstrate that *Acidaminococcus fermentans* ferredoxin/flavodoxin-NAD^+^ reductase (Rnf) activity is dependent on the concentration of Na^+^ ions. Apart from protein identification, MALDI-TOFMS has been used for identification of a variety of reaction product(s) (Chowdhury et al., 2014; Bai et al., 2017). Recently, the methods, which are capable of providing information on protein structure, dynamics, and connectivity in its native environment (including *in vivo* studies) have become a primary choice. These techniques include chemical cross-linking coupled to mass spectrometry (XL-MS), native MS (NMS), surface labeling coupled to MS (SL-MS), hydrogen/deuterium exchange coupled to MS (HDX-MS), as well as protein modeling. Individually, each technique offers a unique way to examine FBEB enzymes, and, when combined, these techniques can be used to obtain detailed in solution structural information revealing key insights into the enzymes’ mechanisms based on the structure–function relationship. The advantage to in solution studies is the ability to gain information about how a protein reacts to various conditions, such as ligand binding or product formation. While X-ray structures are high resolution, they are only snapshots of a protein’s most stable conformation in crystallization conditions where as in solution studies allow for the determination of a protein’s conformation over time while in its native environment (Nogales, 2015).

## Chemical Cross-Linking Coupled to Mass Spectrometry

**FIGURE 1:**
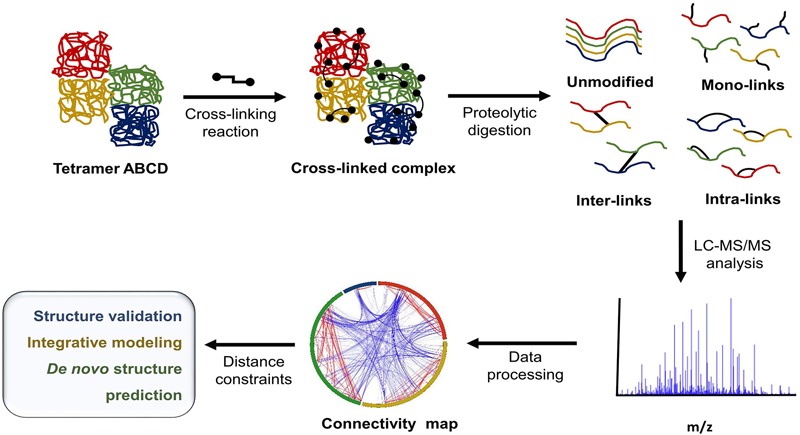
Typical XL-MS workflow. In its simplest form, the XL-MS protocol requires four steps: (1) conversion of non-covalent interactions within or between proteins in their native state into covalent bonds, (2) proteolytic digestion of the cross-linked sample, (3) liquid chromatography - tandem mass spectrometry (LC-MS/MS) analysis of generated peptide fragments, (4) identification of cross-linked peptide species and their linkage sites. The information about spatial/distance constraints can be used further in structural validation, integrative modeling, or *de novo* structure prediction.

In recent years, the growing need for alternative tools for protein structural characterization and protein network identification has led to the development of multiple variations of XL-MS protocols. XL-MS relies on the introduction of a covalent bond between two spatially proximal amino acid residues by a chemical reagent. These artificially fixed interactions are capable of surviving denaturing conditions and can be analyzed using methods that normally would be destructive to non-covalent interactions. This allows for the elucidation of dynamics within a single protein, including conformational changes ranging from rigid body movements to fine tuning through allosteric regulation. XL-MS also allows for low-resolution characterization of multimeric protein complexes, detection of transient interactions, and identification of low affinity interaction partners. In recent years technological advancement and rapid development of software tools have greatly increased the extent of *in vitro* and *in vivo* applications ranging from simple structure validation through integrative modeling to *de novo* structure prediction (Young et al., 2000; Herzog et al., 2012; Kaake et al., 2014; Fernandez-Martinez et al., 2016) and whole proteome studies (Liu et al., 2015; Zhong et al., 2017). **Figure 1** outlines a typical XL-MS experiment.

The selection of a cross-linking reagent is a compromise between the number of generated cross-links and spatial accuracy. With a longer spacer arm (the chain that makes up the cross-link backbone and connects the reactive moieties), more connections can be generated, however, information on inter- or intra-protein interaction sites will be less precise. Therefore, a comprehensive analysis often requires application of cross-linking reagents with different lengths of spacer arms that also target different amino acid residues. Targeting basic and acidic residues is attractive since collectively they are prevalent and well distributed across solvent-accessible protein surfaces. The most commonly used cross-linking reagents are homobifunctional reagents containing N-hydroxysuccinimidyl ester active groups (NHS) at either end of a spacer arm, for example, disuccinimidyl suberate (DSS or its water soluble analog BS3, 11.4Å spacer arm). Heterobi(tri)functional reagents are also available. One of the most commonly used is the zero-length cross-linker, 1-ethyl-3-(3-dimethylaminopropyl)carbodiimide (EDC), which conjugates two residues without adding any spacer arm atoms. This unique property enables formation of the shortest connection (peptide bond) between a proximal lysine residue and an EDC-activated aspartic or glutamic acid residue (Fernandez-Martinez et al., 2016). Another class of widely used heterobifunctional reagents includes compounds containing an amine-reactive NHS ester group and a photo-reactive moiety, such as diazirine, phenyl azide, or benzophenone. Respective examples from each group are as follows: N-succinimidyl *p*-benzoyldihydrocinnamate (SBC, 10.2Å spacer arm) (Krauth et al., 2009); succinimidyl-diazirine (SDA, 3.9Å spacer arm), azido-benzoic-acid-succinimide (ABAS, 7.0Å spacer arm), carboxy-benzophenone-succinimide (CBS, 7.0Å spacer arm), 4-(sulfosuccinimidylcarboxy)benzophenone (SBP, 5.7Å spacer arm) (Brodie et al., 2015; Belsom et al., 2017). The incorporation of a photo-reactive group with non-specific reactivity permits the bridging of proximal residues in hydrophobic regions, thus extending XL-MS application beyond just the structural determination of charged, solvent-accessible interactions (Suchanek et al., 2005). More specialized cross-linking reagents can be equipped with moieties of specific functionality, such as reagents with affinity tags like biotin, which allow for the enrichment of cross-linked species within a complex peptide mixture (Trester-Zedlitz et al., 2003; Petrotchenko et al., 2011). However, the use of these compounds can be limited due to their size and chemical properties, which can affect ionization and peptide fragmentation during downstream analysis.

To investigate protein dynamics and conformational flexibility, a special class of isotopically coded cross-linking reagents was developed (Müller et al., 2001; Petrotchenko et al., 2005, 2011). This innovative concept relies on reacting two conformers with a light and heavy version of a cross-linking reagent. Then the two samples are mixed in 1:1 ratio before being analyzed by LC-MS. This mixing step allows for the direct comparison of the abundance of cross-links in different conditions. Because the cross-linkers differ only in mass, peptides containing different labels have the same retention time and ionization efficiency. This makes it possible to directly infer abundance from the intensity of co-eluting ions. Thus, comparisons of the relative abundances of light and heavy labeled cross-linked peptide pairs enables a determination of the relative abundance of cross-linkable states under different experimental conditions. The addition of deuterium is the most common isotope used, but other stable isotopes can be incorporated into the cross-linker as well (Fischer et al., 2013; Schmidt et al., 2013).

A breakthrough for XL-MS applications was the development of cleavable cross-linking reagents. These reagents contain labile bonds sensitive to photo- (Yang et al., 2010), chemical- (Petrotchenko and Borchers, 2010), and MS-induced cleavage (Tang et al., 2005; Kao et al., 2011; Petrotchenko et al., 2011). Specifically, the introduction of reporter ions (part of the spacer arm with specific fragmentation properties inside the mass spectrometer) significantly improved identification of cross-linked peptide species (only these peptides which contain a reporter ion will be subjected to further analysis). Thus decreasing data complexity and shortening analysis time. These combined benefits make MS-cleavable cross-linking reagents the most attractive type of cleavable reagents for XL-MS studies.

The XL-MS protocol is particularly valuable because it allows for the simultaneous determination of a protein’s identity, dynamics, and connectivity in its native environment. This often leads to the generation of highly complex data caused by several factors. First, protein motion and conformational change can mean that there is an ensemble of conformers in solution. Second, vibrations/rotations within the cross-linker spacer arm cause the spacer arm to have a variable length distribution rather than just one, fixed conformation. Third, cross-linking reactions produce several types of linkages, which provide different depths of spatial and structural information, for example: (i) inter-subunit cross-links (usually the least abundant form) deliver information on overall complex shape and long-distance interactions; (ii) intra-subunit cross-links provide information on a more local scale such as secondary structure; (iii) dead-end cross-links, or mono-links, provide insight into solvent accessibility, especially areas that lack interactions or connectivity with other amino acid residues.

Due to the uneven distribution of amino acids in the protein sequence, cross-linking data is scattered. In some cases, the same peptide sequence might be found in more than one location within a protein or complex leading to some ambiguity in data interpretation. Also, due to the attachment of a chemical modification, not all cross-linked peptides will be detected. In mass spectrometry methods, a lack of data is usually assumed to be inconclusive. However, if the same observation applies to groups of linkages in the same area, it can suggest that a lack of data is a result of a real event (protein related) rather than a random process.

**FIGURE 2:**
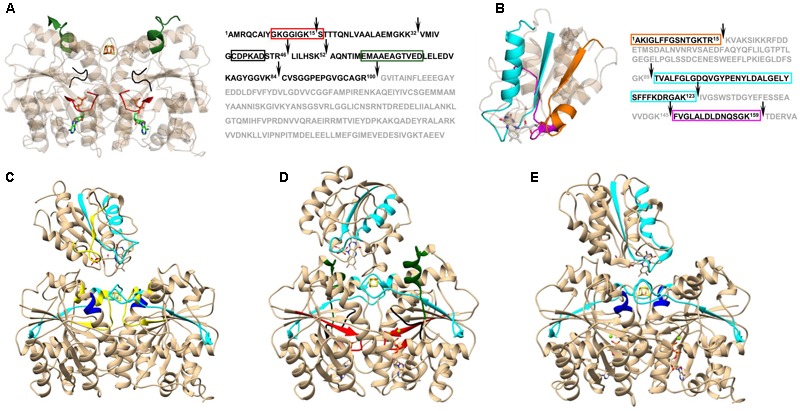
Nucleotide induced differences in *Azotobacter vinelandii* Iron Protein (FeP) and Flavodoxin (Fld) interactions observed in limited proteolysis and cross-linking patterns. Time-resolved limited proteolysis reactions revealed differences in FeP-Fld interaction that were dependent on the nucleotide status of the FeP. The main change observed in FeP upon nucleotide binding **(A)** was associated with the P-loop (red) and the Switch I region that coordinates the bound Mg^2+^ of the nucleotide (black), as well as the Switch I region responsible for MoFe protein binding interface (green). Overall, complex formation with Fld had a minor effect on FeP structure, however, nucleotide induced structural rearrangements in FeP determined the final fit and interaction area with Fld **(B)**. Fld protein regions directly involved in interactions with FeP are highlighted in cyan (near FeP active site), magenta (FeP Switch II), and orange (at the Lys170 of FeP). Cleavage sites in limited proteolysis experiments are indicated with black arrows. The cross-linking patterns revealed the main difference between free **(C)** and nucleotide bound FeP **(D,E)** in the Switch II region (yellow), which was reported to be directly involved in Fld binding (Yang et al., 2016). Differences between MgATP- **(D)** and MgADP-bound **(E)** FeP occurred in the P-loop (red), both Switch I regions (black and green), and in a peripheral Fld binding site at Lys170 of FeP (dark blue). Protein regions were mapped on corresponding protein complex structures acquired by docking of the following Protein Data Bank templates: 2NIP (nucleotide free FeP), 4WZB (MgAMPPCP-bound FeP), 1FP6 (MgADP-bound FeP), and 1YOB (Fld). Figure modified from Pence et al. (2017).

On its own, XL-MS does not usually provide sufficient information to create a structural model with high confidence. Nonetheless, when combined with other surface-probing protocols such as oxidative foot printing (Mummadisetti et al., 2014; Baud et al., 2016), SL-MS (Song et al., 2015), HDX-MS (Liu et al., 2016; Zanphorlin et al., 2016), and limited proteolysis (Birolo et al., 2016; Pence et al., 2017) it can be successfully applied to study a protein’s structure, as well as its dynamics and conformational changes. An example of the information gained through the combination of XL-MS and limited proteolysis is displayed in **Figure 2**. Based on changes in proteolysis and chemical cross-linking patterns, Pence et al. (2017) were able to determine distinct Iron Protein (FeP) regions that are involved in structural differences due to nucleotide binding and complex formation with its physiological reductant, flavodoxin (Fld). FeP is part of the two component catalytic molecular machinery called molybdenum-dependent nitrogenase found in diazotrophs, such as *Azotobacter vinelandii*, responsible for nitrogen fixation. The nitrogenase FeP cycle involves transient associations between the reduced, MgATP-bound FeP and the MoFe protein and includes electron transfer, ATP hydrolysis, release of P_i_, and dissociation of the oxidized, MgADP-bound FeP from the MoFe protein (Hageman and Burris, 1978). One of the major findings was that differences in MgATP-bound FeP were consistent with nucleotide-induced structural differences of FeP in the MgAMPPCP stabilized nitrogenase complex (Tezcan et al., 2005). Further analysis of the proteolytic patterns revealed that a subset of FeP-Fld interactions are maintained (presence of the cross-link) only when FeP is in MgADP-bound state. This suggests that Fld binding to FeP is dependent on the nucleotide form and provides a mechanism for driving the catalytic cycle forward. Since Fld favors interactions with MgADP-bound FeP, whereas MoFe has a higher affinity for the MgATP-bound FeP, the electron delivery to MoFe is directed by a conformationally driven association and disassociation process (Pence et al., 2017).

## Protein Surface Labeling Coupled to Mass Spectrometry

**FIGURE 3:**
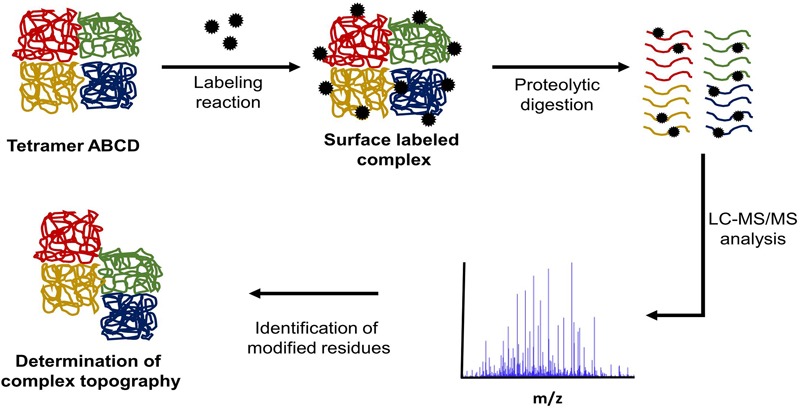
Typical SL-MS protocol workflow. In a SL-MS experiment, there are four major steps: (1) label the protein of interest with the chosen labeling reagent, (2) quench the labeling reaction at multiple time points, (3) digest the labeled protein samples with the chosen protease (protease of choice often targets different residue than labeling reagent), (4) LC-MS/MS analysis of the generated peptide fragments and subsequent identification of modification sites.

Another method of in solution protein structure analysis is surface labeling coupled to mass spectrometry (SL-MS). This protocol is based on the concept that solvent exposed regions of a protein will react more quickly with labeling reagent than regions buried inside the protein core or protected by ligand binding (Suckau et al., 1992; Glocker et al., 1994). **Figure 3** outlines a typical SL-MS workflow. This technique can also be applied to protein complexes involved in transient interactions to determine protein–protein interfaces. For each protein in the complex, two labeling reactions are conducted. The first reaction labels each monomeric protein in solution while the second one labels the assembled protein complex. The labeled peptides are then compared and the differences in labeled regions show which peptides are most-likely involved in forming protein–protein interfaces within the complex (Steiner et al., 1991). This same conceptual approach can be applied to identify both cofactor and ligand binding sites (Roeser et al., 2010).

Following the quenching of the labeling reaction, proteolytic digestion of the protein is required. The choice of protease becomes particularly important when labeling reagents target the same amino acid residues as those, which are required for enzyme proteolytic activity. Using a protease with high specificity allows for the generation of a theoretical digest *in silico*, which in turn allows for a list of expected peptide masses to be compiled. By analyzing the sample by mass spectrometry and comparing the detected peptides to the list of peptide masses from the *in silico* digest, the modified peptides are identified. This process is carried out using protein and peptide analysis platforms such as SearchGUI/PeptideShaker (Vaudel et al., 2015), MaxQuant (Cox and Mann, 2008), or Scaffold (Searle, 2010). These programs identify mass shifts, which correspond to the mass of the modification created by the labeling reagent. When these data are compiled from the multiple time points along the surface labeling reaction time course, it becomes possible to identify which protein regions are the most solvent exposed.

**FIGURE 4:**
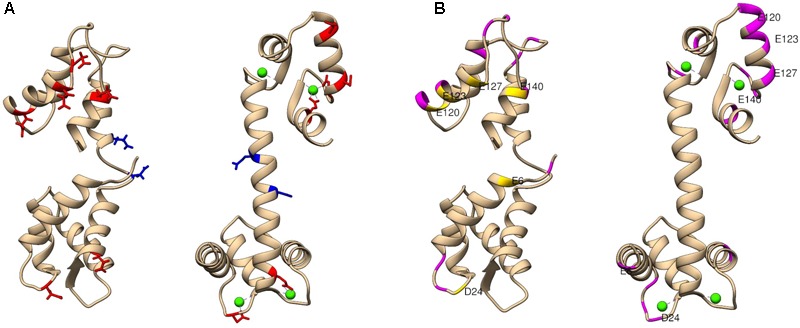
The extent of glycine ethyl ester (GEE) modification as detected by LC-MS/MS for calcium-free and calcium-bound calmodulin (CaM). **(A)** Shows residues labeled to a greater extent in apo CaM (left) in red and residues labeled to greater extent in holo CaM (right) in blue. For context, blue and red residues are highlighted in both structures. **(B)** Shows CaM regions of low label intake (less than 10%) in both conformations in magenta. Additional residues with low label load present only in apo CaM only are colored in yellow. A potential explanation for less exposure of the surface residues can be that calcium-free CaM has a flexible structure in solution, and its central linker region can be bent to accommodate different relative positions of the N- and C-terminal domains. PDB identifier: for calcium-free (1CFC) and calcium-bound (1CLL) calmodulin used in these studies. Figure adapted from the study performed by Zhang et al. (2012).

Labeling reagents come in a wide variety of forms and functionalities. The chemistry of the selected labeling reagent determines the specific type of structural data produced. For example, use of glycine ethyl ester (GEE) covalently modifies the side chains of glutamic and aspartic acids, which facilitates mapping of protein surface carboxyl groups (Zhang et al., 2012; Wecksler et al., 2015). Dansyl chloride (DnsCl) covalently modifies the side chains of lysine and serine residues. DnsCl is particularly powerful among surface labeling reagents, as it not only has a high specificity and rapid reaction rate, but it is fluorescently active as well. Including a fluorescent moiety in the labeling reagent structure allows for SL-MS experiments to be coupled with supplementary analyses such as tracking of protein unfolding in solution (Hsieh et al., 2014), or protein dynamics using fluorescence resonance energy transfer (Kim et al., 2013). The EDC-activated GEE labeling of solvent accessible carboxyl groups of glutamic and aspartic acid residues was successfully used to probe conformational changes in calmodulin upon binding of calcium ions (Zhang et al., 2012) presented in **Figure 4**. Calmodulin is a small protein that has two main domains, which bind calcium by electrostatic interactions to two EF-hand motifs (helix-loop-helix regions) in each domain. These ET-hand motifs are rich in negatively charged residues. Since the GEE labeling reaction rate is slower than protein folding or unfolding, HDX-MS was used as a protein integrity evaluation method and confirmed that there is negligible conformational change during the carboxyl-group modification of calmodulin.

While there are numerous labeling reagents that operate through specific residue modification such as GEE and DnsCl, there are also non-specific surface labeling techniques. One of the most prevalent of these techniques is called oxidative footprinting. This approach relies on ultra-short oxidation of protein surface residues and subsequent identification of modification sites by LC-MS/MS. The oxidation of the protein surface is conducted using oxygen-radical species, which are typically generated by: (i) oxidative Fenton chemistry (Ermacora et al., 1992; Grunberg et al., 2012); (ii) fast photochemical oxidation (Zhang et al., 2011); and the most popular currently (iii) water radiolysis using gamma rays or high energy electrons (Sclavi et al., 1998). Further methods of oxidative footprinting include a method called “stability of proteins from rates of oxidation” or SPROX. This method uses hydrogen peroxide to produce non-specific protein surface oxidation maps (West et al., 2008). In this reaction, 14 of the 20 common amino acids can be oxidized (Wang and Chance, 2011). A major advantage of oxidative foot printing is its reliance on the use of a small chemical probe (rather than a bulky label), which can typically be generated in close to physiological conditions. Also, oxidative footprinting can be applied on a microsecond timescale, making it perfect for investigation of transient interactions between proteins and small molecules.

One caveat of SL-MS involves the concept of label load. As greater numbers of label are incorporated into a protein over time, it becomes progressively less favorable for the protein to maintain its native fold. Another caveat is that when comparing the surface mapping of a monomer to the surface mapping of the complex itself, the possibility that the monomer adopts a different conformation when assembled within the complex should be taken into account. Finally, it should be noted that the structural data produced by this method, while unique, can be relatively limited, as there are only so many labels which can be incorporated into a protein before the protein’s native state becomes compromised.

Advantages of the SL-MS technique include versatility, specific or random amino acid targeting, and its ability to be coupled with other structural analysis methods through use of reagents with added functionality. An underappreciated advantage is that the SL-MS protocol can deliver high-quality output of comparable resolution to HDX-MS and XL-MS methods but with significantly less data analysis required. This data reduction is a double-edged sword since complete protein labeling is impossible to achieve. However, full protein coverage is not often required allowing for the power and versatility of SL-MS to become apparent.

## Native Mass Spectrometry

**FIGURE 5:**
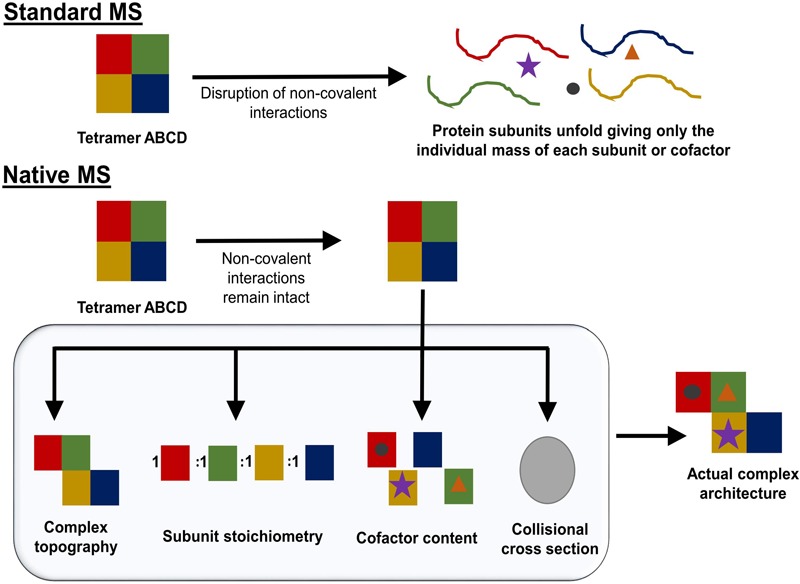
Schematic overview of what can be accomplished using NMS. While standard mass spectrometry methods interrupt the native conformation of the protein complex being studied **(Top)**, native mass spectrometry can retain non-covalent interactions **(Bottom)**. By gradually increasing the amount of energy used in the NMS experiment, complex topography, subunit stoichiometry, and cofactor content can be determined. If the mass spectrometer used has the capability to perform ion mobility, the complex’s collisional cross-section can be determined. Taken together, this information can give an estimation of a protein complex’s architecture.

Native mass spectrometry (NMS) is a form of mass spectrometry in which all native contacts within a protein or protein complex are maintained during analysis in the mass spectrometer. This means the process of NMS must maintain all non-covalent interactions that are present in solution during transition from the liquid to the gas phase, the ionization of the protein, and the transfer through the instrument to the detector. NMS works by increasing the pressure within the instrument thus allowing for the dispersion of energy from the ion to an inert gas (collisional cooling). Collisional cooling reduces the likelihood of breaking non-covalent interactions and increases the transfer efficiency through the instrument for large, compact molecules (Chernushevich and Thomson, 2004). The preservation of the protein fold decreases the amount of surface charge that can be added during the ionization process (Uetrecht et al., 2010). Charge state envelopes can therefore be used to assess folding/unfolding. By using this technique, a protein or a protein complex’s structure and intermolecular interactions can be probed to determine protein complex stoichiometry, topography, cofactor content, as well as a general idea of potential protein conformations (**Figure 5**; Kirshenbaum et al., 2010; Laganowsky et al., 2013).

**FIGURE 6:**
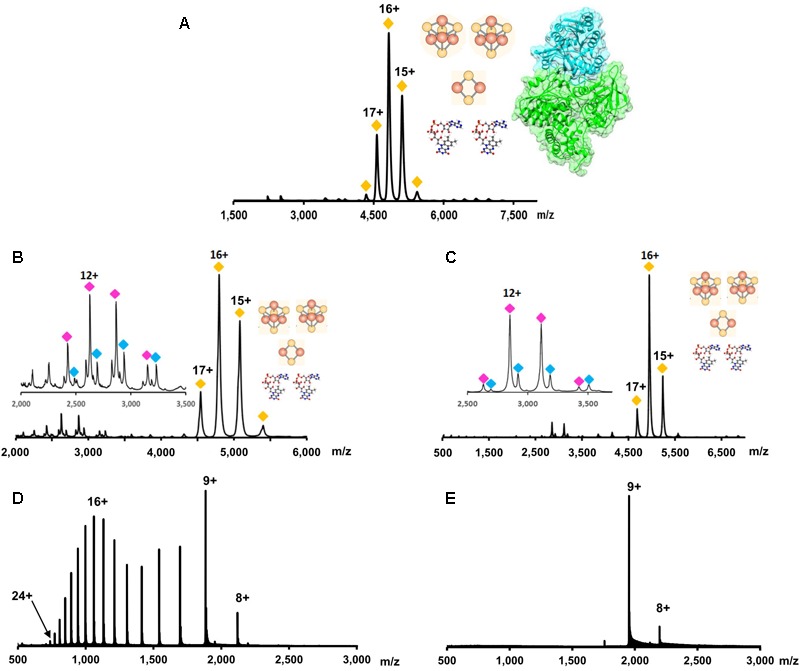
Native mass spectrometry data for the determination of protein complex stoichiometry, cofactor content, and conformation. **(A)** The overall complex stoichiometry and cofactor content for NfnI from *P. furiosus* was determined using NMS. Yellow diamonds indicate the charge state envelope of the complete NfnI complex with two FADs, two [4Fe-4S] clusters, and one [2Fe-2S] cluster. Figure adapted from Berry et al. (2018b). **(B)** It was determined that a single FAD was associated with the smaller of the two subunits in NfnI. Purple and blue diamonds indicate the charge envelope of the small subunit without and with a FAD cofactor, respectively. **(C)** Same as **(B)**, but data are for the protein complex NfnII. **(B,C)** adapted from Nguyen et al. (2017). **(D)** Typical charge state distribution for a denatured protein (e.g., myoglobin). In the presented spectrum, there are two apexes, which suggest a bimodal distribution. This represents the protein in two conformations; at lower m/z values the conformation is more extended (denatured) while at higher m/z values the protein is more compact (native). **(E)** Charge state distribution for myoglobin analyzed in native conditions. A more compact protein structure allows for less charge on the protein surface causing a lower charge state and a more narrow charge state distribution.

Because non-covalent interactions are maintained while the complex is transferred into the gas phase, the molecular mass of the complete complex can be determined. This molecular mass can include all subunits and cofactors that make up the functional, *in vivo*, complex. The advantage of NMS is shown by Berry et al. (2018b) where the *Pyrococcus furiosus* Nfn complex composition in solution was confirmed to be a structurally complete complex in which the subunit stoichiometry and cofactor content matched the published crystal structure (two protein subunits and five cofactors: two FAD, two [4Fe-4S] clusters, and one [2Fe-2S] cluster) (**Figure 6A**; Lubner et al., 2017). *P. furiosus* is a hyperthermophilic archaeon, which utilizes variety of carbohydrates and peptides to produce acetate, carbon dioxide, hydrogen and in presence of elemental sulfur, hydrogen sulfide. The Nfn complex is thought to be involved in maintaining the cellular redox by balancing three pools of redox cofactors: NADPH, NADH, and Fd during carbohydrate metabolism.

Once the composition of the holoprotein complex is determined, NMS can then be used to determine subunit composition and stoichiometry. This occurs through the dissociation of the complex while in the gas phase. As more energy is added to the protein complex, less stable subunits (maintaining the least number of inter-complex interactions) will denature (Sciuto et al., 2011). Denaturation of a subunit will disrupt its interactions with the rest of the protein complex and cause the subunit to be ejected from the complex (Sciuto et al., 2011). By analyzing which subunits are ejected from the complex at lower energies compared to subunits ejected from the complex at higher energies, observations about the strength of protein–protein interactions and general complex topography can be made (Schmidt et al., 2013). Using this technique, Jore et al. (2011) determined the general topography for a 500 kDa protein complex called Cascade. Once a crystal structure of Cascade was available, the general connectivity of the protein subunits within the complex proved to be similar to those proposed by NMS (Jackson et al., 2014).

As protein subunits are denatured and ejected from the complex, the interactions between the remaining protein subunits and their cofactors can be retained. Thus, detection of a cofactor upon subunit ejection can be used to reveal to which subunit a cofactor is bound. Using this technique Nguyen et al. (2017) determined the cofactor content of the subunits of both NfnI (**Figure 6B**) and NfnII (**Figure 6C**) from *P. furiosus*, which each contain two subunits and the same set of five cofactors: two FAD molecules, two [4Fe-4S] clusters, and one [2Fe-2S]. NfnII is a paralog of NfnI, which plays a key role in regulation of redox homeostasis. Despite similarities to the NfnI complex in primary sequence and overall protein and cofactor composition, both enzymes are differentially expressed depending on sulfur availability and the type of carbon source while also having different electron bifurcation potentials. In addition, it was proposed that the FAD cofactors are bound to the Nfn enzymes with different affinities. In the gas phase, the FAD cofactor from large subunit dissociated easily, even at low collision energy conditions (just enough energy to maintain ion transfer within mass spectrometer without breaking non-covalent interactions), while ejection of the second flavin molecule, from the small subunit, required additional energy, **Figures 6B,C** inset (Nguyen et al., 2017).

Information about protein conformation can also be ascertained using NMS. Because all non-covalent interactions are maintained, protein secondary and tertiary structures are also retained. When the charge state distribution of a protein from an NMS experiment is compared to the same protein’s mass spectrum from a standard MS experiment, the charge state distribution can help determine the protein’s fold. The more extended the protein’s conformation, the higher the average charge on the protein and the broader the charge state distribution (Uetrecht et al., 2010; Sciuto et al., 2011; **Figure 6D**). The opposite will be seen in a more compact folded protein (**Figure 6E**).

Native mass spectrometry capabilities can be further extended through ion mobility spectrometry (IMS). While NMS provides information on the mass and charge of a protein, IMS uses drift time (the time it takes an ion to traverse a drift cell) to determine the protein’s shape or collisional cross section (CCS) (Dugourd et al., 1996; Mortensen et al., 2017). While various instruments accomplish this measurement using different physical properties, in modern drift cells, the amount of time it takes an ion to traverse a drift cell filled with an inert collision gas, such as nitrogen, is determined. Larger ions collide more often with the collision gas and, therefore, take more time to traverse the cell. By measuring the drift time, an estimation of the collisional cross-section can be made (Uetrecht et al., 2010; Konijnenberg et al., 2013).

Comparison of collisional cross sections between proteins in different states allows for the study of protein conformations and their relative populations under variable conditions. Studies have shown that the collisional cross sections determined using NMS-IMS are very similar to the cross sections predicted using X-ray crystallography (Smith et al., 2007). With this technique, it is possible to detect changes in CCS as small as 5% (Uetrecht et al., 2010). Capitalizing on this ability, Alexander et al. (2013) showed that using NMS-IMS, an unfolding profile of either wild type or mutant protein dimer can be probed, and the general stability of the different dimers can be compared. This comparison was performed by monitoring the change in CCS over an increasing range of collision energies, which caused the proteins to unfold over time. Proteins that started unfolding at lower energies were deemed less stable than those that unfolded at higher collision energies. NMS is a powerful technique for looking at protein–protein interactions, and the level of detail that can be obtained with NMS is only improved when combined with the other in solution techniques discussed in this review.

## Protein Modeling

When a crystal structure is not available, protein modeling can be used to develop a statistically probable protein structure if the amino acid sequences of the protein are known. Protein modeling began by using an *ab initio* approach, which calculates the potential chemical interactions possible within the amino acid sequence (Hardin et al., 2002). Because every potential interaction needs to be considered in an *ab initio* approach, modeling proteins larger than 100 amino acid residues is a computationally intensive and time-consuming endeavor (Ovchinnikov et al., 2017). Once the possible chemical interactions have been determined, the lowest energy folding events or conformations are chosen as the most likely solution to a protein’s native structure (Hardin et al., 2002). The calculation time that it takes to determine a protein’s structure can be lowered through the combination of *ab initio* modeling with other protein structural prediction techniques.

Within nature it does seem that the number of possible protein sequences is infinite, however, the number of protein folds present in biology appears to be limited to less than 10,000 (Koonin et al., 2002). This limitation may occur through evolution because a protein fold is more highly conserved over time than a protein’s primary amino acid sequence (Koonin et al., 2002). Owing to the highly conserved nature of protein folds, it is possible to lessen the amount of time and computation needed to predict a protein structure by basing the unknown protein’s fold on the fold of homologous proteins.

There are many template-based approaches that compare a queried protein’s primary sequence to a list of known protein structures and then work to overlay the sequence onto the structure that is most well conserved among homologs. Of these tools, I-TASSER, ROBETTA, HHpred, RaptorX, MODELLER, IntFOLD, and Phyre2.0 are all run from web-based servers and are well known for producing model predictions that closely resemble the crystal structure once solved (Kryshtafovych et al., 2017). The readily available online programs seem to have very similar accuracy in their predictions, but Phyre2.0 is the most user-friendly version for researchers who are not familiar with protein modeling (Kelly et al., 2015).

**FIGURE 7:**
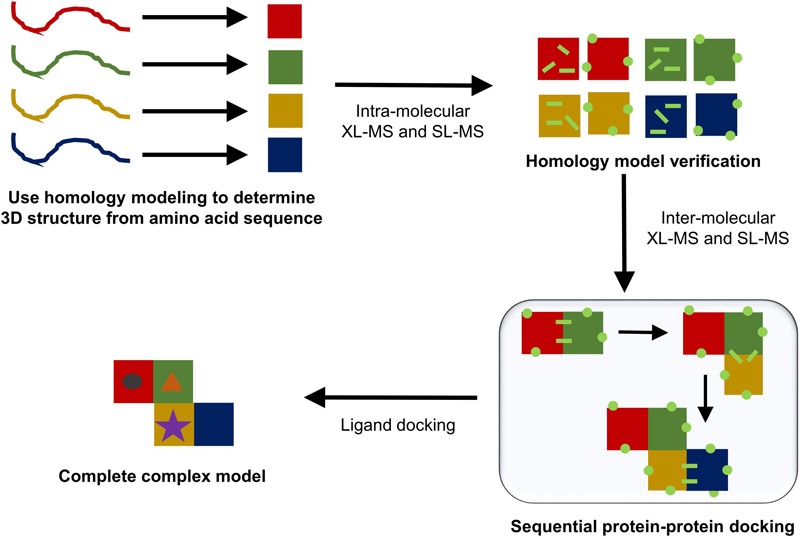
Typical protein modeling workflow. In solution techniques can be combined with each step of the modeling process (homology modeling, sequential protein–protein docking, and ligand docking) to help either validate a presented model or to aid in choosing one model over the others.

Homology modeling generally determines the most likely 3D structure of a protein by first performing a standard sequence alignment against sequences of proteins with known protein structures. The alignment generated will work best if there are a high number of homologous proteins with solved structures and if these homologous sequences show significant diversity (Kelly et al., 2015). Once the alignment is complete, standard methods are used to perform a secondary structure prediction for the protein based on its amino acid sequence. This secondary structural prediction is then matched to homologous proteins with known folds, which are used to determine the overall tertiary structure of the protein. Places where a sequence may differ due to either insertions or deletions into the amino acid sequence are then modeled in using either a series of known short amino acid residue motifs or an *ab initio* approach. Lastly, the side chains are modeled into the structure and any side chain clashes are solved using the known possible angles for side chain rotamers (Kelly et al., 2015). The end product of the template-based homology modeling approach produces a PDB file that can be used in the place of a protein crystal structure. This produced structure can then be further verified using in solution techniques like XL-MS and SL-MS. **Figure 7** gives a schematic outline of the steps taken to produce a potential model for a complete protein complex structure.

While homology models can be quick and relatively easy to generate, there are a few downsides to using this approach instead of the more computationally intensive protein folding simulation tools. This approach will not work if the primary amino acid sequence does not show strong homology with known protein structures (Kelly et al., 2015). Furthermore, the fewer homologous structures that can be found to compare a submitted sequence to, the more the produced homology model will differ from the protein’s actual structure. While homology modeling can predict the local effects of a point mutation, it is well known that point mutations may show strong allosteric effects within a protein, which cannot be predicted using a homology modeling approach (Packianathan et al., 2010). Finally, the current tools cannot use homology modeling to predict the structure of multimeric complexes.

Many structural or enzymatic activities within a cell are achieved by oligomers of proteins rather than individual monomeric proteins. This leads to a need for structures of protein complexes. While homology modeling cannot yet predict the overall architecture of these multimeric structures, coupling homology modeling with protein docking tools can give a likely model for a complex. ClusPro2.0 uses a thermodynamics-based approach to determine which protein–protein interactions between two submitted protein structure files fall in the lowest energy well (Kozakov et al., 2017). It achieves this by first performing rigid-body docking in which one protein is held static and the other samples billions of conformations around the static structure. Of those billion conformations, the 1,000 lowest energy conformations are then grouped so that conformations that are similar are placed together. The most populated groups are then chosen as the most-likely candidates and go through further refinement and energy minimization. The calculations used by ClusPro2.0 to determine the energy of protein–protein interactions are based on four different energy parameters in a standard search: balanced, electrostatic-favored, hydrophobic-favored, van der Waals, and electrostatics. From there, the top 10 models for each parameter are returned to the user. This produces 40 models that are all similar in probability. Because these models can differ significantly from one another, in solution techniques such as XL-MS and SL-MS can be used to further reduce the number of possible models. These data can even be incorporated directly into the modeling process by either acting as a restraint (XL-MS data) or an attraction/repulsion (SL-MS data) during protein–protein docking (Kozakov et al., 2017).

Once a dimeric protein model is made, if the complex is thought to contain additional subunits, proteins can be docked onto the multimeric structure one at a time using the above approach. This method is limited by the size of the complex; once the complex becomes too large, this docking approach is no longer feasible (Kozakov et al., 2017). Chimera can be used to visualize the complex once the model is generated, and the Chimera plugin Xlink Analyzer can be used to determine the validity of the proposed models based on cross-linking data (Pettersen et al., 2004; Kosinski et al., 2015). This plugin will provide statistics for satisfied cross-links as well as enabling the user to manually manipulate the subunit positioning to further refine the docking performed by modeling tools (Kosinski et al., 2015).

If the cofactor content of a complex is confirmed by another technique, online tools such as SwissDock can be used to determine the position of cofactors within the protein. SwissDock uses similar energy minimization techniques as the other modeling programs discussed to determine the lowest energy potential binding sites of a small molecule ligand within a submitted protein structure (Grosdidier et al., 2011).

**FIGURE 8:**
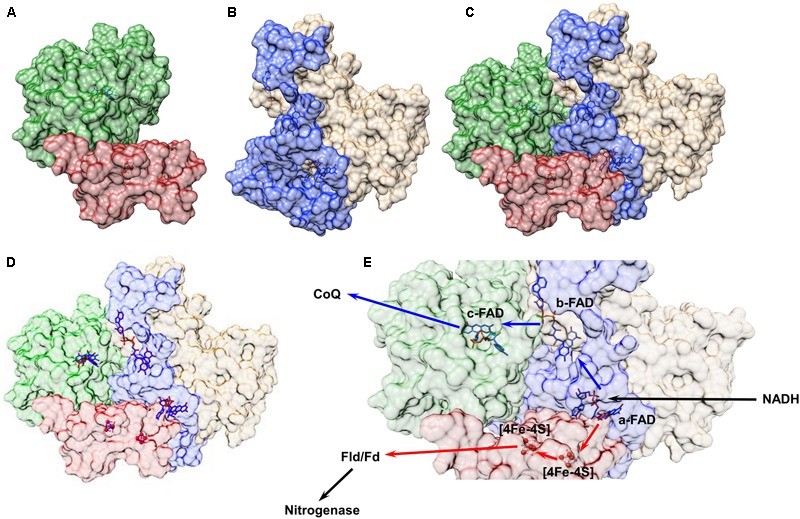
Structural model of the FixABCX complex from *A. vinelandii* as determined by a combination of molecular modeling and chemical cross-linking. All protein structures were created using Phyre2.0. Chemical cross-linking data plus the homology models were input into ClusPro2.0 to establish the protein–protein interactions between subunits C (green) and X (red) **(A)** and subunits A (blue) and B (tan) **(B)** of the Fix complex. Each dimer was then uploaded to ClusPro2.0 and docked using cross-linking data as a restraint to generate the complete complex shown in **(C)**. The protein cofactors, FAD and [4Fe-4S] clusters, were then modeled into the complete quaternary complex structure using SwissDock **(D)**. Based on the distances between cofactors a plausible electron transfer pathway in the FixABCX system could be proposed **(E)**. The bifurcation process begins at the a-FAD (in FixA), which accepts a pair of electrons from NADH and directs one to coenzyme Q (exergonic branch represented by blue arrows) through flavins in FixB and FixC, and the other electron to Fld/Fd (and eventually to FeP in the nitrogenase complex) through the low-potential [4Fe-4S] clusters in FixX (endergonic branch represented by red arrows). Figure was adapted from Ledbetter et al. (2017).

In Ledbetter et al. (2017), all of these techniques – homology modeling, protein–protein docking, and protein–ligand docking – were used to develop a low-resolution model of the FixABCX protein complex from *A. vinelandii* (**Figure 8**). In the cell, the FixABCX system bifurcates electrons from NADH to coenzyme Q (high-potential acceptor) and Fld/Fd (low-potential acceptor) in order to provide electrons to FeP nitrogenase in support of nitrogen fixation. Since *A. vinelandii* requires oxygen for growth, unlike the other bifurcating systems containing [Fe-S] clusters, FixABCX is less oxygen sensitive. Also, this is a first characterized FBEB enzyme, which is membrane associated. To isolate such a system from cell lysate requires additional care to maintain a proper fold of the hydrophobic domains, now exposed to solvent, and to keep the native state of the entire protein assembly (Jakobsson et al., 1999; Carpenter et al., 2008; Ledbetter et al., 2017). The combination of molecular modeling with mass spectrometry based in solution techniques allowed for the determination of the protein–protein interactions within the complex while NMS coupled with biochemical and electrochemical assays confirmed cofactor content and potential cofactor placement (Ledbetter et al., 2017). Because protein structure is closely correlated to protein function, the general idea of the FixABCX complex’s structure allowed for the proposal of plausible pathways for electron transfer.

## Hydrogen/Deuterium Exchange Coupled to Mass Spectrometry

Hydrogen/deuterium exchange (HDX) is a powerful technique that can reveal detailed information about a protein’s dynamics. Protein dynamics influence how a protein or protein complex undergoes enzymatic reactions. Therefore, understanding a protein’s dynamics can reveal information about the connection between structure and function of a protein complex. HDX works by using the ability of peptide amide hydrogens to freely exchange with hydrogens in solution to determine changes in a protein’s conformation during the various steps of a catalytic cycle (Engen, 2009; Konermann et al., 2011; Percy et al., 2012). Other hydrogens within a protein exchange at a rate that is either too fast or too slow to detect using this method (Konermann et al., 2011). By replacing water (H_2_O) with deuterated water (D_2_O), the amide hydrogens will exchange with the deuterons. A time course of a protein in a deuterated solution is used to determine and compare the dynamics of a protein in different conditions. As more deuterons are incorporated into a protein or protein complex, the molecular weight will also increase. When coupled with a mass spectrometer (HDX-MS), how much deuterium is incorporated onto an intact protein, or, if a proteolytic digestion step is performed, how much deuterium is incorporated onto a peptide can be determined (Berry et al., 2018a). When performing peptide level HDX-MS, the resolution of the data is determined by the number of overlapping peptides, which can show individual amino acid contributions to deuterium incorporation (Pascal et al., 2009).

**FIGURE 9:**
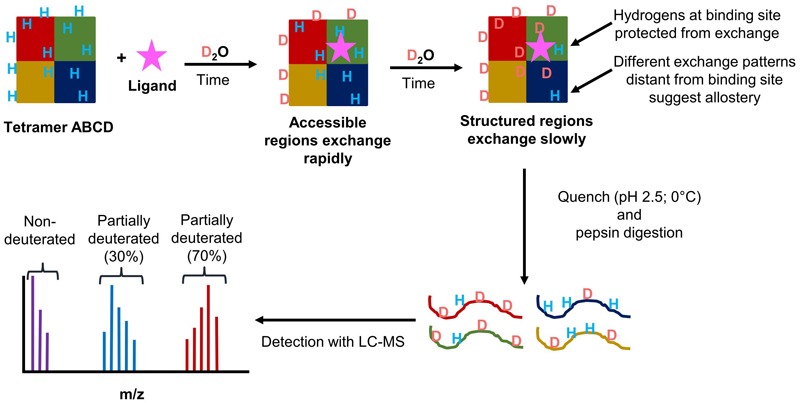
Schematic overview of the workflow of HDX-MS experiments. (1) A theoretical heterotetramer complex in solution with amide hydrogens is exposed to deuterium. (2) Amide hydrogens exchange with deuterons. Solvent accessible regions exchanging rapidly. (3) Protected or structured regions exchange at a slower rate. Protection can occur through ligand binding, protein–protein interactions, or stabilization of secondary structure. (4) At designated time points, the protein is placed in a quench solution containing pepsin to produce deuterated peptides. (5) The peptides are analyzed by LC-MS. From the mass spectrum, the isotopic distribution for each peptide is determined. The three distributions shown represent the non-deuterated (purple), partially deuterated (blue), and almost completely deuterated (red) forms of a detected peptide.

The rate of deuterium exchange is influenced by the structure of a protein of interest. Highly static secondary structures will shield amide hydrogens from exchange, whereas more unstable secondary structures that go through frequent local folding/unfolding events will expose amide hydrogens to exchange (Rumi-Masante et al., 2012; Engen et al., 2013). Tertiary and quaternary interactions can also influence the rate of exchange. Based on a protein’s fold, some residues will be accessible on the surface of the protein while those on the interior of the protein will not be accessible to exchange. With protein complexes, protein–protein interactions will also decrease the accessibility of amide hydrogens to exchange. This is useful for looking at subunit organization in protein complexes. This same concept can also be applied to examining protein–ligand interactions to determine the site of binding, as well as the effects ligand binding can have on the rest of the protein or protein complex. **Figure 9** outlines the general scheme of a HDX-MS experiment.

For HDX-MS, the biggest disadvantage is back exchange. While the exchange of deuterium with an amide hydrogen is a spontaneous reaction, amide deuterons are capable of exchanging with other hydrogens or deuterons in the solvent. While the exchange reaction is occurring, deuterium is the dominant species in the deuterated reaction solution. During the reaction, any exchange after the initial change from hydrogen to deuterium will result in deuterons swapping places. However, during the LC separation prior to MS analysis the sample is introduced to a mobile phase consisting of water plus 0.1% formic acid, which supplies hydrogen atoms that can then back exchange with amide deuterons. The rate of amide hydrogen exchange is dependent on the pH and temperature of the reaction, with pH having the greatest impact on the rate of exchange (Walters et al., 2012). LC solvents typically include formic acid to provide protons for ionization, improve separation of polar compounds, and also because keeping the pH low helps to prevent back exchange. The minimum exchange rate occurs at a pH of 2.5 at 0°C (Walters et al., 2012).

One advantage of using HDX-MS is its ability to be automated. For instance, when a quench-flow apparatus is used to run the exchange reaction, the time scale is no longer limited by an individual’s pipetting skills (seconds to hours), but is instead limited by the quench-flow apparatus (milliseconds to hours). Transient interactions between a protein and ligand or two component proteins typically occur on the millisecond time scale or faster (Coales et al., 2010; Keppel and Weis, 2013). With quench-flow HDX, the location and effects of the transient interactions on a target protein can be observed as they occur. This level of detail is crucial for understanding the role of conformational changes in ligand binding and protein–protein interactions.

**FIGURE 10:**
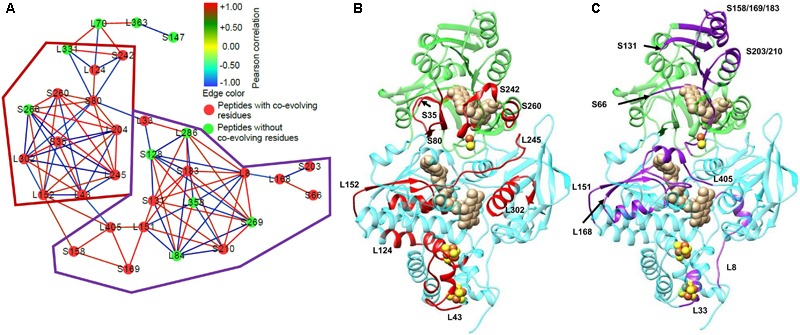
Network analysis of HDX-MS data and co-evolving residues in *P. furiosus* Nfn. Prior to integration with SCA, it was known that communication occurred within Nfn, but the actual mechanism remained elusive. The integration of SCA with the HDX-MS data allowed for the full visualization of the communication pathway within Nfn. **(A)** Peptides detected with HDX-MS represent the nodes, which are colored based on the presence (red) or absence (green) of co-evolving residues identified with SCA. Each of the nodes are connected by edges representing the correlation of the deuterium uptake in the nucleotide or Fd bound conditions (NAD^+^, NADPH, NADPH+NAD^+^, and Fd_Ox_) relative to the nucleotide or Fd free condition. Red edges represent positive correlation between peptides in the nucleotide bound conditions, whereas blue edges represent negative correlation between peptides. Based on regions with high connectivity between peptides, sub-networks of similar features were circled by the red and purple outlines in **(A)**. To better understand how Nfn facilitates communication between the two subunits, peptides with co-evolving residues were mapped onto the 3D structure of Nfn. **(B)** The red sub-network includes protein regions near cofactors binding sites: the S-FAD and the [2Fe-2S] cluster (S35, S80, S242, S260), L-FAD, and the 2[4Fe-4S] clusters. **(C)** The purple sub-network features protein regions near cofactors and nucleotides binding sites: the NAD^+^/NADH binding site (S131, S158/169/183), S-FAD and the [2Fe-2S] cluster (S66, S203/210), and L-FAD (L151, L168, L405). After localizing the peptides with co-evolving residues, further investigation of the network edges, and therefore the correlation of exchange, reveals which peptides are communicating with one another when positive correlation is present. The goal of this analysis is to identify areas of communication within a protein complex to characterize mechanisms of allosteric regulation. Figure adapted from Berry et al. (2018b).

The ability of HDX-MS to compare the differences in protein conformation in different conditions has begun to be used in studies on electron bifurcating enzymes. Initial studies used HDX-MS to look at ligand binding in the *Thermotoga maritima* Nfn complex (Demmer et al., 2016) and the effects of ligand binding on the dynamics of the *P. furiosus* Nfn (Lubner et al., 2017). These studies were capable of using the differences in deuterium incorporation in either the presence or absence of NADPH in order to determine its binding location, and the effects of binding on structural dynamics. Additionally, these studies identified the first evidence of allosteric regulation in the Nfn complex, opening the door to more intensive studies of Nfn. One such study combined HDX-MS with the bioinformatics technique statistical coupling analysis (SCA) to identify networks of communication within the complex (**Figure 10**; Berry et al., 2018b). SCA is an increasingly popular technique for looking at the co-variation in amino acid residues using a multiple sequence alignment. By examining the amino acid composition in multiple sequences, co-evolving residues can be identified. If there is a correlation of amino acid substitution between two positions in a protein sequence, these residues are considered to co-evolve with one another (Lockless and Ranganathan, 1999; Halabi et al., 2009). SCA reveals pathways of communication between co-evolving residues in a protein or protein complex. This study found high correlation between the ligand and cofactor binding sites, suggesting multiple mechanisms of allosteric regulation of the bifurcating and confurcating reactions (reaction reverse to bifurcation) based on which ligands are bound to the complex.

## Conclusion

Due to the high complexity of FBEB enzymes, probing the details of the electron bifurcation mechanism is impossible using any single approach, even such superior ones as X-ray crystallography, cryo-EM, or NMR. While these methods offer exceptional resolution, in-solution studies provide deep insights into protein dynamics and conformational flexibility. Specifically, in solution protocols combined with the sensitivity of MS detection offer considerable analytical power. In addition, the small sample amount required to perform a comprehensive analysis along with the lack of a protein size limitation, very little optimization and, in some cases near-amino acid resolution, make in solution MS-based methods particularly attractive. Taking advantage of both static and dynamic approaches in addition to *in silico* simulations is key for understanding how electron bifurcation and electron gating defines FBEB.

## Author Contributions

All authors listed have made a substantial, direct and intellectual contribution to the work, and approved it for publication.

## Conflict of Interest Statement

The authors declare that the research was conducted in the absence of any commercial or financial relationships that could be construed as a potential conflict of interest.
